# News and Coming Events

**Published:** 1909-02-06

**Authors:** 


### NEWS AND COMING EVENTS.

The King has been pleased to appoint Mr. George Andreas Berry, M.B., C.M. Edin., F.R.C.S. Edin., to be honorary surgeon-oculist to his Majesty in Scotland, in the room of the late Dr. Argyll Robertson.

The addresses to be delivered at the next annual meeting of the British Medical Association have been arranged as follows: The Presidential Address will be delivered by Sir William Whitla, M.D., LL.D., Professor of Materia Medica and Therapeutics, Queen's College, Belfast; the address in Medicine by Frederick Taylor, M.D., F.R.C.P., Consulting Physician, Guy's Hospital; the address in Obstetrics by Sir John W. Byers, M.D., Professor of Midwifery and Diseases of Women, Queen's College, Belfast; and the Popular Lecture by J. A. Macdonald, M.D., M.Ch., Physician to the Taunton and Somerset Hospital, chairman of the representative meetings.

The annual report of Livingstone College is by far the most satisfactory report which has been issued since the College was founded fifteen years ago. The number of students who entered is easily the largest on record, being an average of 30 for each of the three terms Livingstone College exists for giving elementary medical training, and it has no denominational or national prejudices. The working expenses of the year have been practically met by the fees of students, showing that if a sufficiently large number of resident students enter for a full session the College might be self-supporting. The great need of the College is a regular supply of students to take advantage of the facilities which are there afforded for elementary medical training. Contributions will be gratefully acknowledged by the principal, Dr. C. F. Harford, Livingstone College, Leyton, E., who will also gladly send full particulars concerning the various courses of training.

Dr. J. E. Gordon has been appointed to the post of medical superintendent of the Devon and Cornwall Sanatorium.

The first of the Lettsomian Lectures for 1909 at the Medical Society of London was delivered by Dr. Sidney Martin, F.R.S., on February 1. The remaining two lectures will be delivered on February 15 and March 1, at 9 p.m. on each evening. The subject of the lectures is "Functional Disorders of the Stomach and Intestines, their Diagnosis from Organic Disease, and Treatment."

The Princess of Wales, on behalf of the National Hospital for the Paralysed and Epileptic, Bloomsbury, has consented to receive purses in aid of the Jubilee Fund some time during the autumn. Over 500 collections are being made for this Fund (some of them by late patients and by pensioners of the hospital) in all parts of the United Kingdom. The amount aimed at by the Jubilee Fund is £50,000, of which £10,000 is to be devoted to the extension of the premises, a commencement of the building having just been made.

Metropolitan Asylums Board.—At the last ordinary meeting of the managers of the Metropolitan Asylum District, in reply to a letter from the Metropolitan branch of the Incorporated Society of Medical Officers of Health, it was decided to inform that society that, in the event of the Local Government Board deciding to add puerperal fever to the list of infectious diseases for which the managers are required to provide accommodation, the managers would be prepared to make arrangements for the reception of certified cases into the hospitals under their control.

Dr. George Ellis, said to be the oldest Irish surgeon, died recently at his residence in Dublin at the age of 100 years. He took the B.A. degree of the University of Dublin in 1831, the M.B. degree in 1834, and became a Fellow of the Royal College of Surgeons in Ireland in 1844, after obtaining the license in 1837. He was the author of "Irish Ethnology, Socially and Politically considered," and contributed several articles to medical journals. In one of these articles he recorded the benefit he had derived from a remedy, which he prepared and called "Rennet Wine," for gastric derangements of the stomach. Dr. Ellis retired from practice forty years ago.

A meeting of the Senate of the University of London was held on January 27, the Vice-Chancellor (Sir William Collins, M.D., M.P.) being in the chair. Mr. G. H. Cowen, M.B., B.S., was appointed member of the Council of Hartley University College, Southampton, and Mrs. Scharlieb, M.D., M.S., Governor of St. Mary's College, Paddington. Miss Muriel Robertson was also appointed to be one of the assistants to the Professor of Protozoology in place of Dr. J. D. Thomson, resigned; and a letter was received from Dr. P. H. Pye-Smith, M.D., F.R.C.P., F.R.S., intimating his resignation of his membership of the Senate as one of the representatives of the Royal College of Physicians of London.

At the last *Comitia* of the Royal College of Physicians of London, held on Thursday, January 28, the President, Sir Richard Douglas Powell in the chair, the following Fellows of the College were elected Councillors: Sir William Allchin, in the place of the late Dr. C. E. Beevor; Dr. Sidney Phillips, Dr. W. Pasteur, Dr. S. Martin, and Dr. A. E. Garrod, in the place of Dr. W. Osler, Dr. Crocker, Dr. Tooth, and Dr. Acland, who retired by rotation. Dr. F. H. Champneys was re-elected to represent the College on the Central Midwives Board; and Dr. C. Theodore Williams was elected a representative on the Court of Governors of the University of Birmingham.

Mr. A. G. R. Foulerton, F.R.C.S., has been appointed Milroy Lecturer at the Royal College of Physicians of London for 1910, and the Swiney Prize of the College has been awarded to Dr. C. A. Mercier, F.R.C.P., for his work, "Criminal Responsibility."

The annual meeting of the Italian Hospital was held at the hospital, Queen Square, on Saturday, Count Bosdari (Councillor of the Italian Embassy) presiding, in the unavoidable absence of the Italian Ambassador. Among those present were Lord Edmund Talbot, M.P. (vice-chairman of the committee), the Marquis di Bruno, Cav. Dr. Vincent Dickinson, and Mr. G. L. Cheatle, C.B. The report states that 9,545 patients were treated during 1908, of whom 802 were in-patients. They comprised an appreciable number of British subjects besides members of other nations. The annual subscriptions showed a small increase. An encouraging feature of the year's receipts was the new support which the hospital had received from Italians resident in Italy. The Chairman stated that the committee could always rely on the sympathetic interest of the Italian Embassy in London. Lord Edmund Talbot, M.P., made a sympathetic reference to the recent earthquake, and said that the deepest sorrow was felt in this country at the terrible catastrophe which had befallen the Italian people. The report was adopted.

